# Improvement of Intestinal Immune Cell Function by Lactic Acid Bacteria for Dairy Products

**DOI:** 10.3390/microorganisms5010001

**Published:** 2016-12-23

**Authors:** Tomonori Kamiya, Yohei Watanabe, Seiya Makino, Hiroshi Kano, Noriko M Tsuji

**Affiliations:** 1 Biomedical Research Institute, National Institute of Advanced Industrial Science and Technology (AIST), Tsukuba, Ibaraki 305-8566, Japan; t-kamiya@aist.go.jp (T.K.); yohei-watanabe@aist.go.jp (Y.W.); 2 Food Science Research Laboratories, Meiji Corporation, 540 Naruda, Odawara, Kanagawa 250-0862, Japan; seiya.makino@meiji.com (S.M.); hiroshi.kano@meiji.com (H.K.)

**Keywords:** Lactic acid bacteria, Peyer’s patches, Dendritic cells, T helper cells, *Lactobacillus bulgaricus*, *Streptococcus thermophilus*, Interferon γ, Interleukin 17, Mucosal immunity, Probiotics

## Abstract

Lactic acid bacteria (LAB) form a major component of gut microbiota and are often used as probiotics for fermented foods, such as yoghurt. In this study, we aimed to evaluate immunomodulatory activity of LAB, especially that of *Lactobacillus bulgaricus* ME-552 (ME552) and *Streptococcus thermophilus* ME-553 (ME553). In vivo/in vitro assay was performed in order to investigate their effects on T cell function. After oral administration of ME553 to C57BL/6 mice, the amount of both interferon γ (IFN-γ) and interleukin 17 (IL-17) produced by cluster of differentiation (CD) 4^+^ T cells from Peyer’s patches (PPs) were significantly enhanced. On the other hand, ME552 only up-regulated the production of IL-17 from PP cells. The extent of induction for IFN-γ production differed between ME552 and ME553. These results suggest that LAB modulate T cell effector functions and mucosal immunity.

## 1. Introduction

Lactic acid bacteria (LAB) are well known as commensal in gastrointestinal tract of any animalia, and typical probiotic bacteria utilized in various types of fermented foods [1,2]. For more than ten thousand years, LAB have contributed to improve flavor, preservation, and nutrition of fermented foods represented by pickles, yogurt, soy sauce, and fermented soybean pastes. Moreover, it is indicated that these fermented foods promote health through modulation of multi-dimensional biological mechanisms, such as immunological and neurological function [3]. In fact, several LAB are utilized even in the clinical settings [4,5]. Nevertheless, the beneficial effects of LAB may differ among strains and detailed evaluation should be made for each strain of LAB of interest upon industrial application. 

Recent studies revealed that intestinal bacteria interact with hosts’ mucosal immunity and modulate the functional maturation of various immune cells. Both foreign microbes and host commensal microbes contact the surface of the body and contribute to build tissue-specific immunity in the skin or gastrointestinal tract [6,7,8,9]. Several bacteria are shown to affect mucosal immunity, including T cell differentiation, innate immune activity, and antibacterial activity. Segmented filamentous bacteria (SFB) are members of commensal microbes in the small intestine of mice [10,11]. It has been shown that SFB enhance the induction of T helper 17 cells and are suggested to relate to autoimmune symptoms in disease-model mice. For another example, some kinds of *Clostridium* species induce colonic regulatory T cells and result in suppressing inflammatory bowel disease in a mouse model [12]. We have reported that double-stranded RNA of LAB stimulate Toll-like receptor 3 and induce interferon-β, which protect mice from colitis [13]. Another study showed that lipoteichoic acid from *Lactobacillus acidophilus* regulates colonic inflammation induced by dextran sulfate sodium [14]. In both cases there are innate immune receptors that recognize the molecules in LAB and enhance anti-inflammatory mechanisms. 

Yoghurt became famous by the finding of Elie (Ilya) Mechnikov, who claimed consumption of LAB-containing yoghurt relates to the prolongation of life. *Lactobacillus bulgaricus* and *Streptococcus thermophilus* are well-known LAB and starters of yoghurt with great industrial value [15]. Recently, new types of yoghurt fermented with any kinds of LAB or *S. thermophilus* are also produced as popular dairy products. Previous studies showed these probiotic strains regulate inflammation, suggesting their ability to modulate the function of immune-related cells [16,17,18,19,20]. The aim of this study is to provide information and clarify the characteristics of yoghurt-derived LAB strains for immune-modulatory effects, especially on the activation of dendritic cells (DCs) and T helper (Th) cells in the intestine. Immune recognition of bacteria components through pattern recognition receptors (PRRs) in DCs depends on organic compounds no matter if bacteria are alive or dead. In fact, it is assumed that there are a fair amount of dead bacteria in fermented food and the intestine from gut microbiota. Here we demonstrate how the effects of bacterial components from killed LAB potentially stimulate mucosal immunity.

## 2. Materials and Methods 

### 2.1. Mice

C57BL/6 mice were purchased from Sankyo Lab Service (Tokyo, Japan). OT-Ⅱmice, with a transgenic background for T cell receptors that recognize ovalbumin (OVA) peptide, were bred at AIST. Eight to 10 week old female mice were used in the present study. All procedures using mice were reviewed and approved by the Institutional Care Use of Animals Committee of AIST and were performed according to Guidelines for Animal Use and Experimentation of AIST including the provisions of animal ethics (animal experiment number 109 and 111). 

### 2.2. Lactic Acid Bacteria

*Lactobacillus bulgaricus* ME-552 and *Streptococcus thermophilus* ME-553 were cultured and heat-killed at Meiji holdings Co., Ltd (Tokyo, Japan). ME552 and ME553 were stationary cultured in MRS or M17 broth for 18 h at 37 °C. Bacteria were harvested, washed twice, and re-suspended in sterile saline and the suspensions were then heated for 1 h at 75 °C (heat-killed) and were stored at −30 °C until use. Each product lot was tested whether these heat-killed cells form colonies after the process. The amount of LAB for the purpose of daily oral administration to a mouse is 1 × 10^9^ cfu/200 μL saline.

### 2.3. Cell Isolation from Peyer’s Patches (PPs)

PPs were collected from small intestines to a dish containing 2 mL of RPMI1640 (Wako, Tokyo, Japan) and 2% fetal calf serum (FCS) (Thermo Fisher Scientific K.K, Kanagawa, Japan) (RPMI + 2), and washed with RPMI + 2. Then PPs were transfered to a new dish containing intestinal epithelial cell (IEC)-dissociating solution (RPMI1640, 10% FCS, 25 mM 4-(2-hydroxyethyl)-1-piperazineethanesulfonic acid (HEPES), 5 mM ethylenediaminetetraacetic acid (EDTA), 1 mM dithiothreitol (DTT)), and incubated still in the incubator for 45 min at 37 °C in a 5% CO_2_ condition. Next, IEC were dissociated from PPs by vigorous pipetting, and PPs were transfered to a new dish containing RPMI1640, 10% FCS, 5mM EDTA, and further incubated for 5 min at 37 °C in a 5% CO_2_ condition. After incubation, PPs were washed with RPMI + 2 in the dish and transfered to the digestion solution (RPMI1640, 10% FCS, 400 U/mL Collagenase type I (Sigma-Aldrich, Missouri, United States), 60 U/mL DNase I (Roche)). Tissues were incubated for 30 min at 37 °C with stirring, then the cell suspension were collected. Remaining tissues were subjected to the second digestion with the same conditions. When the second digestion was completed, tissues were passed to the new 50 mL conical tube through a cell strainer with 40 μm pores. Cells from the first and second digestion were combined and cultured in the medium (RPMI1640 (Thermo Fisher Scientific K.K) with 10% FCS and 1% penicillin-streptomycin, in the presence or absence of anti-CD3ε (145-2C11) and CD28 (PV-1) antibodies (2 μg/mL, each)), and supernatants were collected after 48 h.

### 2.4. Isolation of CD11c^+^ Cells

CD11c^+^ cells from PPs or spleen were purified to >90% by positive selection using CD11c^+^ microbeads and a MACS LS column (Miltenyi Biotec; Bergisch Gladbach, Germany) according to the manufacturer’s directions.

### 2.5. Isolation of Naïve T Cells

CD4^+^ cells from spleen were isolated by negative selection using CD4^+^ cell isolation kit and MACS LD column (Miltenyi Biotec; Bergisch Gladbach, Germany) according to the manufacturer’s directions. Naïve T cells were further purified with PE-conjugated CD62L (MEL-14) antibodies, anti-PE beads, and MACS LS column (Miltenyi Biotec).

### 2.6. Generation of Bone Marrow-Derived Dendritic Cells (BMDCs)

Bone marrow cells were collected from tibiae and femur of C57BL/6 mice by flushing bone cavities with RPMI + 2. After hemolysis, cells were labeled with PE-conjugated anti-CD4 (CK1.5), CD8 (Ly-2), and I-A/I-E (M5/114.15.2) antibodies, and conjugated with anti-PE magnetic beads, and then passed though a MACS LD column (Miltenyi Biotec), depleting all relevant cells. Remaining cells were cultured in RPMI 1640 containing 10% FCS and GM-CSF (20 ng/mL). At day 3, half of the culture supernatant was removed and the same amount of medium containing fresh GM-CSF (20 ng/mL) was added. On day 8, non-adherent BMDCs were harvested and were cultured with LAB.

### 2.7. CD11c^+^ Cells and Naïve T Cells Co-Culture

After spleen cells were smashed using a syringe, CD11c^+^ ( from wild-type mice) or naïve CD4^+^ T cells (from OT- II mice) were prepared as described above. CD11c^+^ cells from spleen or PPs of wild-type mice were pulsed with OVA peptides (0, 50, 250 ng/mL) and co-cultured with naïve CD4^+^ T cells from OT-II mice in the presence of heat-killed *L. bulgaricus* ME-552 or *S. thermophilus* ME-553 for 72 h. The ratio of cells in this co-culture method is as follows: CD11c^+^ cell:naïve T cells:ME552 or ME553 = 1:5:50 (multiplicity of infection to CD11c^+^ cells—MOI:50).

### 2.8. Measurement of Cytokine

Cytokine production was measured by enzyme-linked immunosorbent assay (ELISA), using mouse IL-10, IL-17, and IFN-γ DuoSet ELISA (R and D Systems, Inc., Minneapolis, MN, USA) according to the manufacturer’s instructions. Cytokine concentrations were calculated using the standard curve obtained for each measuring plate. 

### 2.9. RNA Extraction, cDNA Synthesis, and qRT-PCR

RNA was extracted with a commercial kit following manufacturer’s instructions (NucleoSpin RNA: Takara Bio, Shiga, Japan). cDNA was synthesized from total RNA using the PrimeScript™ RT reagent Kit with gDNA Eraser (Takara Bio) according to the manufacturer’s instructions. The quantitative real-time PCR (qRT-PCR) was evaluated using SYBR Premix Ex Taq™ II (Takara Bio) and Thermal Cycler Dice Real-Time System TP800 (Takara Bio) according to manufacturer’s instructions. Primer sequences used are listed in Table 1.

### 2.10. Statistical Analysis 

Differences in data in parametric analysis were evaluated by the Student’s *t*-test. Differences of *p* < 0.05 were considered statistically significant.

## 3. Results

### 3.1. CD4^+^ Cells Activation in Peyer’s Patches by LAB

**Figure 1:**
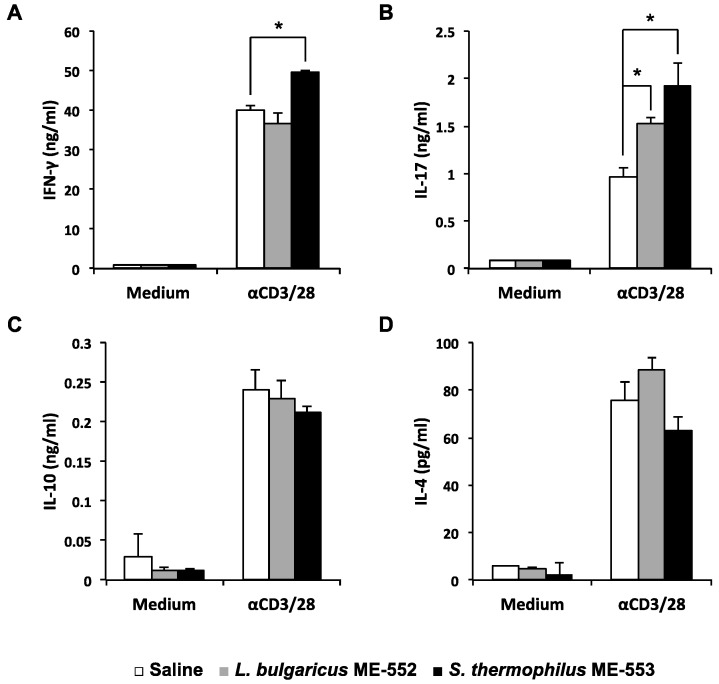
Cytokine production from the Peyer’s patch T cells derived from the mice orally administrated with lactic acid bacteria. Mice were orally administrated with 1 × 10^9^ cfu heat-killed *L. bulgaricus* ME-552 or *S. thermophilus* ME-553 once daily. Lymphocytes from the Peyer’s patches were stimulated with anti-CD3ε and anti-CD28 antibody for 48 h, and the levels of IFN-γ (**A**), IL-17 (**B**), IL-10 (**C**), and IL-4 (**D**) in the supernatant were determined by ELISA. Values are expressed as mean ± SD for three experiments. Different from the saline orally administrated, Student’s *t*-test was performed for *p*-values. * ; *p* < 0.05 vs control.

To understand the immune-modulatory effect by *L. bulgaricus* ME-552 (ME552) and *S. thermophilus* ME-553 (ME553) in vivo, C57BL/6 mice were orally administrated with heat-killed bacteria daily. After seven days, single cell suspensions from the PPs of these LAB-fed mice were prepared, and cultured in the presence of anti-CD3ε and -CD28 antibodies in order to stimulate T cells. In case of ME553, levels of both interferon-γ (IFN-γ) and IL-17 in the culture supernatant were higher compared to control (Figure 1A,B). On the other hand, ME552 enhanced IL-17, but not IFN-γ, which remained at the same level as the control. No significant change was observed in Th2 cell cytokines, i.e., IL-4 and IL-10, in the cell culture (Figure 1C,D).

### 3.2. Antigen-Specific T Helper Cell Differentiation by LAB

**Figure 2:**
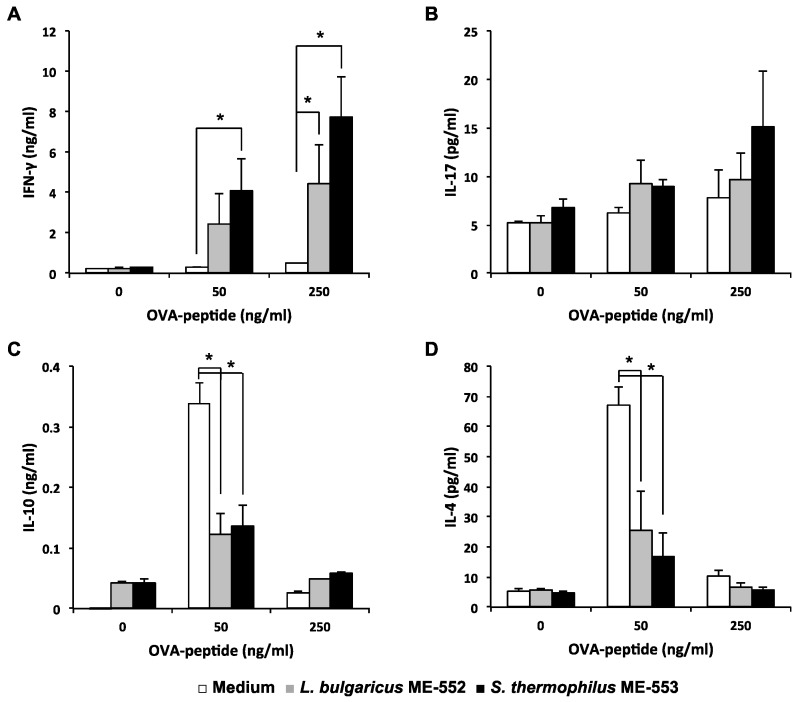
Effects of lactic acid bacteria on T cells response upon antigen-pulsed dendritic cells from spleen. Splenic CD11c^+^ cells from wild-type mice were pulsed with OVA peptides (0, 50, 250 ng/mL) and co-cultured with splenic naïve CD4^+^ T cells from OT-Ⅱ mice in the presence of heat-killed *L. bulgaricus* ME-552 or *S. thermophilus* ME-553 for 72 h. The ratio of cells in the co-culture was as follows: CD11c^+^ cells:naïve T cells:ME552 or ME553 = 1:5:50. Levels of IFN-γ (**A**), IL-17 (**B**), IL-10 (**C**), and IL-4 (**D**) in the supernatant were determined using ELISA. Values are expressed as mean ± SD for three experiments. Different from the medium only, Student’s *t*-test was performed for *p*-values. * ; *p* < 0.05 vs. control.

Next, we performed in vitro culture assay to test if LAB affects antigen (Ag)-specific T cell differentiation. Using co-culture system, naïve T cells from OT-II mice were cultured with OVA peptides and CD11c^+^ cells from wild-type mice as antigen-presenting cells (APCs), in the presence or absence of OVA peptides and heat-killed LAB. Both ME552 and ME553 enhanced Ag-specific IFN-γ production compared to control. Enhancement of IFN-γ production was Ag dose-dependent, where ME553 revealed higher effects than ME552 (Figure 2A), but then Production of IL-17 was hardly observed by this experimental system (Figure 2B).

In contrast, production of Th2 related cytokines such as IL-4 and IL-10 were suppressed in cultures with both LAB (Figure 2C,D). It seems to be a reciprocal result from the higher activity of IFN-γ producing Th1 cells.

### 3.3. Th1 Cell Differentiation with CD11c^+^ Cell from Peyer’s Patches by LAB

**Figure 3:**
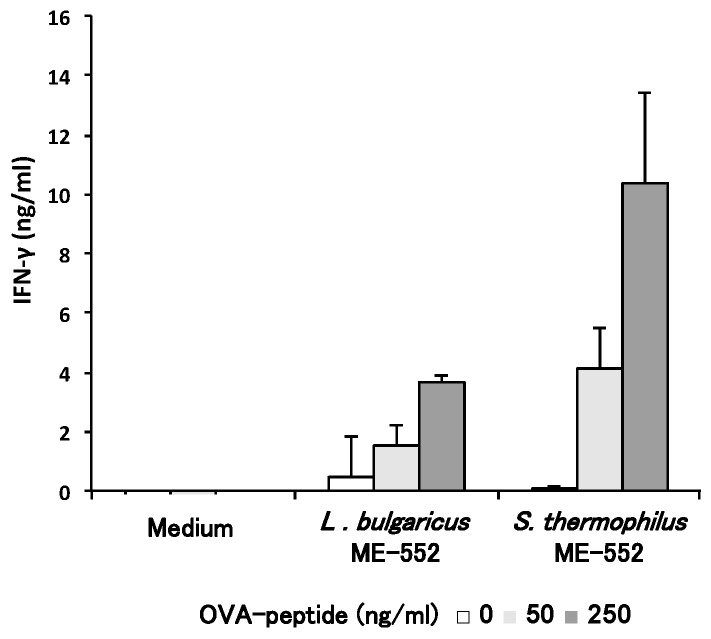
Effects of lactic acid bacteria on T cell response upon antigen-pulsed dendritic cells from Peyer’s patches. CD11c^+^ cells that from the Peyer’s patch of wild-type mice were pulsed with OVA peptides (0, 50, 250 ng/ml) and co-cultured with naïve CD4^+^ T cells from spleen of OT-Ⅱ mice in the presence of heat-killed *L. bulgaricus* ME-552 or *S. thermophilus* ME-553 for 72 h. The ratio of cells in the co-culture was as follows; CD11c^+^ cells:naïve T cells:ME552 or ME553 = 1:5:50. IFN-γ concentrations in the supernatant were determined using ELISA. Values are expressed as mean ± SD for three experiments. Different from the medium only, Student’s *t*-test was performed for *p*-values.

PPs are mucosal lymphoid tissues in the small intestine; therefore, DCs in PPs are important APCs that recognize LAB. With similar experimental methods in Figure 2, we examined the effect of LAB to modulate T cell differentiation, using CD11c^+^ cells from PPs as APCs. Since splenic CD11c^+^ cells induced Th1 type cells (Figure 2A) and LAB enhanced this in vitro Ag-specific response, we focused on IFN-γ production from T cells. Both strains of LAB enhanced the production of IFN-γ in the presence of OVA peptides in a dose-dependent manner (Figure 3) and, as in the case that splenic CD11c^+^ cells were used, ME553 showed more intense effect than ME552. 

### 3.4. Induction of Cytokine in Dendritic Cell by LAB

**Figure 4:**
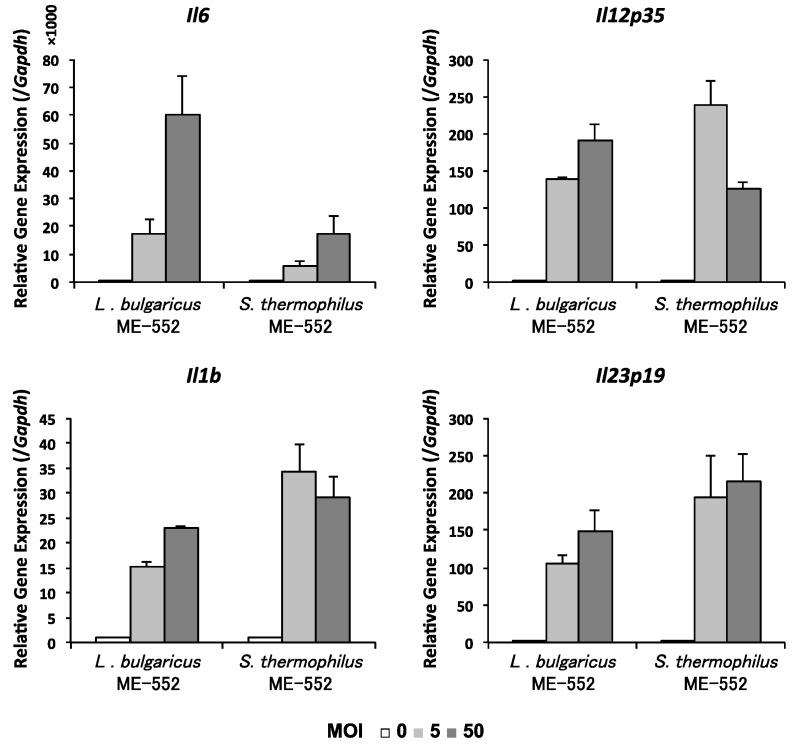
Gene expresson of cytokines in BMDCs treated with lactic acid bacteria. Bone marrow-derived dendritic cells (BMDCs) were culture with heat-killed *L. bulgaricus* ME-552 or *S. thermophilus* ME-553 for six hours, and collected for RNA from BMDCs. The ratio of cells in the co-culture was as follows: CD11c^+^ cell:ME552 or ME553 = 1:0, 5, or 50 (multiplicity of infection—MOI: 0, 5, or 50). qRT-PCR was performed on amplified cDNA to measure gene expression of *Il6*, *Il12p35*, *Il1b*, and *Il23p19*.

Finally, we analyzed if gene expression of DCs are enhanced by LAB, especially those molecules related to T cell differentiation. We utilized bone marrow-derived DCs (BMDCs) stimulated by LAB. As shown in Figure 4, gene expression levels of *Il6*, *Il12p35*, *Il1b*, and *Il23p19* were increased.

## 4. Discussion

Probiotics are bacterial strains that benefit one’s health, and foods containing those beneficial microorganisms. Health-promoting effects of probiotics are brought by improving a balance in intestinal microbiota, together with the effect of activation of mucosal immunity in the gut [13,21,22,23,24,25]. 

Luminal bacteria and antigen molecules are incorporated via M cells into PPs and affect T cell differentiation, which takes place extensively in this tissue [26,27,28]. We found that oral administration of LAB promoted T cell differentiation in PPs (Figure 1). One of Th cell’s potentiality is the amount of cytokine that is produced. The potential ability of given Th cells in the steady state can be evaluated by cytokine production upon activation with anti-CD3 antibodies. We found that the level of cytokine production of IFN-γ and IL-17, which reflect the ability in the steady state of Th1 and Th17 cells, respectively, was up-regulated after oral administration of LAB to mice, indicating protective immunity of those animals was promoted. In PPs, the population of CD4^+^ T cells is about 10% and they are in charge of responding to exogenous Ags to build anti-inflammatory and anti-infectious functions. These T helper cells shows plasticity in their functional properties, including regulatory T cells, which are important for the anti-inflammatory response in the gut, and Th1 or Th17 cells, which are responsible for protective immunity [28,29,30]. Our results indicate that LAB enhance the ability of intestinal T cells to respond to luminal Ags more efficiently. 

Co-culture assay of immune cells was performed to clarify the cause-effect relationship between the LAB recognition by intestinal APCs and up-regulation of T cell activity, monitored by cytokine production. Naïve T cells are best utilized to evaluate the effect of LAB to modify T cell differentiation and function, where antigen dosage is a critical indicater for the determination of these cell propensities [31]. Therefore, different doses of OVA peptides are used in the assay and we confirmed that a low dose of OVA peptide (50 ng/mL) induces IL-4, and IFN-γ is preferentially produced in the presence of high dose of Ag. It is notable that IFN-γ production was highly enhanced by LAB even under low dose of Ag, and the production of Th2 cytokines were significantly suppressed simultaneously (Figure 2). These results indicate that both ME552 and ME553 bacteria, especially ME553, are capable of modulating Th cell differentiation towards Th1, and IFN-γ-producing cells become predominant in the cell culture. This phenomenon may directly relate to the results of in vivo study, in which enhancement of Th1 response, and the increase of Th1/Th2 ratio by the oral administration of heat-killed ME553, indicating the strain may enhance gut protective immunity. Further investigation will be necessary in order to evaluate the physiological effect of ME552, which showed milder effects to ME553 in in vitro study. 

T cell activation and differentiation are initiated and modulated by definite and tissue-specific factors which trigger immune response, such as types of APCs, antigen type or doses, or stromal cells in given tissues [31,32,33,34]. DCs are professional APCs to initiate Th cell polarization. Although the precise mechanisms for DCs to induce functional maturation of T cells are yet to be fully elucidated, expression levels of PRRs in DCs may associate with their ability to activate T cells [35,36]. Our previous study showed that expression of TLR3 is one of key PRRs on DCs upon LAB recognition [13]. 

In the present study we used BMDCs to map the induction of cytokines important for the differentiation of T cells, which may explain how ME553 affect more significantly to skew the Th1/Th2 ratio both in vitro and in vivo (Figure 4). We have reported that both IL-12 and IL-18 are important for enhancing IFN-γ-producing cells by a *Lactoccus lactis* strain [37]. In the present study, we confirmed that both ME522 and ME523 induced *Il12p35* gene expression to promote Th1 cell differentiation. Interestingly, there seems to be a stoichiometric difference between two strains for the induction of *Il12p35*, i.e., ME553 induced higher expression level of the molecule than ME552 under the lower MOI(5) which may better reflect physiological conditions. Future study should cover the production of IL-18, which we revealed to be critical for the effect of LAB to enhance IFN-γ production. 

Meanwhile ME552 induced higher levels of IL-6 expression in BMDC, which promotes IL-17, producing Th17 cells. The results match well with in vivo studies that ME552 enhances Th17 activity. The authors presume that the effect of ME553 to enhance IL-17 production in vivo may be associated with a discrete mechanism. One possibility is a mechanism through IL-1β and/or IL-23 which should be clarified in the future. Enhancement of Th17 activity at mucosal, but not systemic, organs are preferential to protect the body against pathogens, such as fungi [38,39]. Elucidating mechanisms for different propensities between ME552 and ME553 upon regulation of Th17 immunity is another target for future studies. To this end, further investigations on innate immune signals derived from LAB components, in combination with the expression of PRRs on different types of tissue-resident APCs, are underway.

## Figures and Tables

**Table 1:** Quantitative real-time PCR primers sequence.

Gene	Forward (5’-, -3’)	Reverse (5’-, -3’)
*Il1b*	TCCAGGATGAGGACATGAGCAC	GAACGTCACACACCAGCAGGTTA
*Il6*	CCACTTCACAAGTCGGAGGCTTA	CCAGTTTGGTAGCATCCATCATTTC
*Il12p35*	GTCTTAGCCAGTCCCGAAACC	TCTTCATGATCGATGTCTTCAGCAG
*Il23p19*	ACATGCACCAGCGGGACATA	CCTTGTGGGTCACAACCATCTTC
